# Attitudes of primary healthcare nurses towards people living with mental illness in Botswana

**DOI:** 10.4102/sajpsychiatry.v30i0.2316

**Published:** 2024-11-11

**Authors:** Selebogo M. Moremi, Anthony A. Olashore, Philip R. Opondo

**Affiliations:** 1 Department of Psychiatry, Faculty of Medicine, University of Botswana, Gaborone, Botswana; 2 Department of Psychiatry, Perelman School of Medicine, University of Pennsylvania, Pennsylvania, United States of America

**Keywords:** attitudes, mental illness, nurses, primary healthcare, discrimination

## Abstract

**Background:**

The global disease burden attributable to mental and neurological disorders has been increasing over the years. World Health Organization (WHO) recommends the integration of mental health services into existing primary healthcare framework as one strategy for dealing with the burden. Understanding the attitudes of nurses towards people with mental illness is important for a successful integration and management outcome of patients.

**Aim:**

This study aimed to determine primary healthcare nurses’ attitudes towards people with mental illness.

**Setting:**

The study was conducted at Greater Lobatse health district, one of the primary healthcare districts in Botswana.

**Methods:**

A cross-sectional study was conducted among 202 nurses working in the greater Lobatse health district from 01 May 2023 to 30 November 2023. Convenience sampling was used. Data were collected using a structured self-administered questionnaire.

**Results:**

The prevalence of negative attitudes was 51.5%. The mean age (standard deviation [s.d.]) of respondents was 33.4 (8.0) years. Being a non-specialised nurse (*B*= –0.184; *p* = 0.014), having a personal history of mental illness (*B* = –0.215; *p* = 0.002), and having poor knowledge about mental illness (*B* = –0.149; *p* = 0.032) were associated with negative attitudes.

**Conclusion:**

More than half of the respondents have negative attitudes towards people with mental illness. This justifies the need for training and educational programmes and anti-stigma campaigns among primary healthcare nurses to mitigate negative attitudes.

**Contribution:**

This study provides insight into primary healthcare nurses’ attitudes towards people with mental illness.

## Introduction

Mental and behavioural disorders contribute significantly to the global disease burden, with about 16% of disability-adjusted life-years (DALYs).^1^ Mental disorders refer to a range of conditions that can affect one’s behaviour, emotions, and thought processes and can cause distress to the individual or others, impacting social and occupational functioning.^2^ Mental disorders are prevalent worldwide and contribute significantly to morbidity and premature mortality.^3^ Stigma about mental disorders has been identified in existing literature as one of the factors that exacerbate the global disease burden attributable to mental disorders.^4^ Therefore, the World Health Organization (WHO) has recommended various strategies and programmes to address this issue. One of the strategies is integrating mental health services into existing functional primary healthcare systems.^5^ Botswana’s primary healthcare facilities provide first contact for most psychiatric patients and most of these primary healthcare facilities are run by nurses who consult and treat patients.^6,7^

The integration of mental healthcare into primary care settings represents an effective approach to providing accessible, affordable, and holistic treatment. This approach ensures early detection, intervention and follow-up, which can lead to positive treatment outcomes in those with mental disorders. In addition, integrating mental healthcare into primary healthcare reduces the stigma and discrimination associated with specialised psychiatric hospitals.^4^ Nevertheless, the attitudes of healthcare workers have been found to have a negative impact on the delivery of mental healthcare services.^8^ For example, in Botswana, efforts to integrate mental health services into mainstream health systems began in the late 1970s, but faced multiple challenges that mitigated against the intended purpose.^9^

The attitudes of healthcare professionals have been found to affect mental healthcare service delivery^8^ and it refers to a person’s tendency to respond positively or negatively towards an idea, person, object, or situation.^10^ Attitudes of nurses have been found to affect mental healthcare service delivery.^8^ This is particularly concerning, as research has shown that negative attitudes have a negative impact on the management and treatment outcomes of people with mental disorders.^8^

Studies across 20 countries have shown mixed attitudes among nurses towards individuals with mental disorders.^10^ Nurses in Asian and Middle Eastern countries generally hold negative attitudes towards those with mental disorders compared to their counterparts in European countries, who have mixed attitudes.^10^ The divergence in attitudes towards mental disorders across cultures can be partially attributed to the impact of cultural differences and beliefs. Within Asian societies, individuals suffering from mental disorders are often perceived as a menace to society, exhibiting unpredictable and immoral behaviour. Moreover, they may be subjected to punishment for their forebears’ ancestral misconduct.^11^ These negative societal responses often extend to health professionals.^8^

Studies conducted in African settings also show varied attitudes among nurses towards those with mental disorders. Most show significant negative attitudes^12,13,14^ and are because of the fact that most African societies believe that mental disorders are because of familial defects or the result of evil mechanisations leading to stigma against the mentally ill.^15^ In contrast, a few studies conducted in Ghana and South Africa reported positive attitudes of nurses towards people with mental disorders.^16,17,18^ Nonetheless, negative attitudes are a concern in these settings, especially because nurses make up most primary healthcare workers where people with mental illness seek help.^19^

Studies suggest that certain sociodemographic factors, such as being female, higher level of education, being senior staff at work and more years of experience are associated with positive attitudes.^14,20,21,22,23^ To illustrate, two studies reported that female nurses tend to have sympathetic attitudes than male nurses.^20,22^ Nurses with higher levels of education have been reported to have less stigmatising attitudes than those with lower levels of education.^13,14^ This could suggest more theoretical work results in positive attitudes. Senior staff and those with more years of experience of working with general patients tend to exhibit more positive attitudes.^14^ One study revealed that nurses with less than 5 years of experience were 4.84 times more likely to have negative attitudes than those who have work experience of more than 11 years.^14^ More years of experience partially support contact hypothesis, which posits that interaction reduces negative attitude by increasing knowledge and invalidating stereotypes.^24^ Other, factors such as specialised training (in mental health), mental health training, particularly the Mental Health^25^ Gap Action Program (mhGAP), and knowledge about mental illness were associated with the positive attitudes of nurses.^14^ It has been observed that the attitudes of general nurses or those who lack specialised training (in mental health) towards the mentally ill tend to be more negative as compared to those of their specialised counterparts.^25,26^ Given the circumstances, we have hypothesised that negative attitudes may be prevalent in our primary care facility. This is because most of the nurses in our facilities lack specialised training in mental health. For instance, a previous study conducted in the only referral mental health facility in Botswana reported that over 60% of the nurses there were general nurses.^6,27^

Despite their pivotal role in mental health service delivery and a preponderance of unspecialised cadre, as well as the high prevalence of mental health disorders in the region, few studies in Africa have appraised primary healthcare nurses’ attitudes towards people with mental disorders.^19^ As far as our knowledge goes, no studies have yet examined the attitudes of healthcare workers towards individuals with mental illness in Botswana. This knowledge gap poses a significant obstacle to the development of effective mental healthcare interventions. Therefore, the purpose of this study was to determine the attitudes of nurses towards individuals with mental illness and related factors within a primary healthcare context in Botswana. This study’s findings will enable the identification of problematic areas and inform the development of interventions to address these issues.

## Methods and materials

### Study design, sites, and population

The study was conducted in the greater Lobatse Health District, one of several health districts in Botswana, using a quantitative cross-sectional study design. The district comprises 36 primary healthcare facilities, including health posts, clinics, a primary hospital, and a district hospital, and is in the southeastern part of the country. The district’s main offices are situated in Lobatse, a major town in the area that is about 72 km away from Botswana’s capital city, Gaborone. At the time of the study, there were approximately 424 nurses working in the district’s facilities.

The study population encompasses the nursing staff employed in the 36 primary healthcare facilities that operate within the greater Lobatse Health District. As part of the study design, nurses working in private clinics and those on leave during the data collection period were excluded.

### Sample size calculation

The sample size was determined using the Cochran’s formula (1977), using probability of an outcome occurring, which is 48.2%, obtained from a similar study.^14^ Because the population being studied was small, the calculated sample size was further adjusted using a recommended equation.^28^ The final adjusted minimum sample size was calculated to be 202.

### Sampling method

A convenience sampling method was used. This method allowed all nurses who met the inclusion criteria to be potential respondents. However, one disadvantage of this method is that it is prone to selection bias.^29^ In an attempt to minimise sampling bias, questionnaires were distributed across all the different facilities (and departments) that were in the study location to improve sample representativeness. In addition, questionnaires were distributed on various days and times to improve diversity of the sample.

### Data collection procedure

Participants were approached at their convenience in their workstations and were provided with detailed information about the purpose of the study. The consenting nurses working in health facilities were provided with questionnaires to complete. Prior to participating, respondents were assured of their right to withdraw from the study at any time and were informed of their confidentiality and anonymity. The survey was solely administered by the principal investigator, who received and collected the completed questionnaires from the respondents. The principal investigator took necessary measures to ensure the confidentiality of the responses by storing the completed questionnaires in a secure, locked cabinet. Data collection was carried out from May 2023 to November 2023; the period was to allow for coverage of all facilities in the district and to allow those who do shift work and those on leave to participate.

### Measures

#### Sociodemographic questionnaire

A sociodemographic questionnaire was developed based on a review of the literature. It was used to collect all the sociodemographic and clinical characteristics variables. These variables included the respondents’ sociodemographic data, such as their highest level of education, years of experience, and age at their last birthday.

#### Mental illness clinicians attitude scale

Mental Illness Clinicians Attitude Scale (MICA v4) was utilised to measure attitudes, which serve as the outcome variable. It was developed in 2010 by the Health Service and Population Research Department, Institute of Psychiatry King’s College London. It is a 16-item, self-administered tool scored on a 6-point Likert-type scale. Total MICA score is obtained by adding scores for the individual items. Total scores range between 16 and 96 and represent the sum of the individual item scores.^30^ The authors declared that the scale does not have a cut-off point, as it is not easy to claim that there is a level above, which attitudes are negative. It is to be used as a continuous scale, and it is recommended that the mean and standard deviation be used for the cut-off point. A high overall score above the mean indicates a more negative (stigmatising) attitude, whereas a low score below the mean indicates a positive attitude. Items 3, 9, 10, 11, 12, and 16 are scored as follows: Strongly agree = 1, Agree = 2, Somewhat agree = 3, Somewhat disagree = 4, Disagree = 5, Strongly disagree = 6. The remaining items are reverse scored: Strongly agree = 6, Agree = 5, Somewhat agree = 4, Somewhat disagree = 3, Disagree = 2, Strongly disagree = 1.^31^

Cronbach’s value for the MICA-4 scale items was found to be 0.72, indicating a good internal consistency.^31^ The scale has been used in several similar studies across the globe to determine the attitudes of health professionals (including nurses working in primary healthcare) towards mental illness.^14,17,32^

#### Mental health knowledge schedule

Mental Health Knowledge Schedule was used to determine knowledge (stigma-related knowledge) about mental illness. The tool was developed by Thornicroft and his colleague.^33^ This instrument is used in conjunction with other instruments to better understand knowledge related to attitudes and behaviours. It can be self, or interviewer administered. It has 12 items and is scored on a 6-point Likert scale.^33^ The overall internal consistency of items was moderate, with a Cronbach’s alpha of 0.65. However, this value is less important because the instrument was never meant to function strictly as a scale. The instrument is to be used with other instruments. In this study, it was used in conjunction with the Mental Illness Clinicians’ Attitudes Scale (MICA).

Mental Health Knowledge Schedule items are scored on an ordinal scale (1 to 5). ‘Strongly agree’ has a value of 5 points, while 1 point reflects strongly disagree. The total score is calculated by adding individual scores. ‘Don’t know’ is coded as neutral (that is, 3). Items 6, 8, and 12 are reverse coded. Higher total scores correspond to greater knowledge.^33,34^ The tool has been used in several mental health stigma-related studies.^35,36,37^

### Data analysis

The data collected was analysed using the Statistical Package for the Social Sciences (SPSS) version 27. Descriptive statistics such as frequencies were used to describe categorical sociodemographics such as gender and health facility. Clinical characteristics such as personal and family history of mental illness were also summarised using frequencies and percentages. Because the data were normally distributed, mean and standard deviation were used for continuous variables such as age and income, and the dependent variable was the attitude score. Independent *t*-tests were used to test for any association between categorical sociodemographic, clinical factors and the dependent variable, whereas the Pearson correlation was used to explore the relationship between the continuous variables and the outcome.

Those variables that were significant on bivariate analysis at a *p*-value less than 0.05 were further entered into a multiple regression model to test if they were indeed associated with the outcome. A one-step multi-regression analysis was employed to predict the outcome variable, the attitude, based on an array of factors, which included clinical and demographic elements. To prevent the overfitting of the regression model, we utilised the formula *N* > 50 + 8m, where ***m*** equals the number of independent variables, as suggested by Tabachnick and Fidell.^38^ This criterion requires a minimum of 90 cases for five independent variables. Additionally, we ensured that multicollinearity was addressed by maintaining a tolerance value of less than 0.10 and a Variance Inflation Factor (VIF) value of above 10. We considered all associations with a *p*-value of less than 0.05 to be statistically significant.

### Ethical considerations

Ethical approval was sought from the Institutional Review Board of the University of Botswana with ethical clearance number HPRD: 6/14/1, and the Ministry of Health. Greater District Management Health Team gave written permission to conduct the study. Written informed consent was obtained from every participant who agreed to partake in the study. Respondents were instructed not to provide their names or facility names (provided initials instead) on the data collection sheets to ensure anonymity.

## Results

### Sociodemographic characteristics of the respondents

The total number of respondents was 202, the majority being female, 62.4% (126). The mean age (standard deviation [s.d.]) of respondents was 33.4 (8.023) years. Among the total respondents, 79.5% (159) had diploma qualifications, and most of them, 82.4% (164), were general nurses (without specialised training). Table 1 is a representation of the sociodemographic characteristics of the respondents.

**TABLE 1:** Sociodemographic characteristics of 202 primary healthcare nurses.

Characteristic	Statistic			
	Mean	s.d.	Frequency	%
**Age (years)**	33.4	8.023	-	-
**Gender**	**-**	**-**	**202**	**100.0**
Male	-	-	76	37.6
Female	-	-	126	62.4
**Health facility**	**-**	**-**	**202**	**100.0**
Hospital	-	-	113	55.9
Clinic	-	-	88	43.6
Health post	-	-	1	0.5
**Marital status**	**-**	**-**	**202**	**100.0**
Single	-	-	140	69.3
Married	-	-	56	27.7
Separated	-	-	2	1.0
Divorced	-	-	4	2.0
**Religion**	**-**	**-**	**198**	**100.0**
Christianity	-	-	192	97.0
Others†	-	-	6	3.0
**Educational qualification**	**-**	**-**	**202**	**100.0**
Diploma	-	-	159	79.5
Degree	-	-	41	20.5
**Designation**	**-**	**-**	**201**	**100.0**
Registered nurse	-	-	54	26.9
Above registered nurse‡	-	-	147	73.1
**Speciality**	**-**	**-**	**199**	**100.0**
General nurse	-	-	164	82.4
Midwife	-	-	26	13.1
Psychiatric nurse	-	-	6	3.0
Other§	-	-	3	1.5

Note: Bold values indicate the total of participants who responded and frequency.

s.d., standard deviation.

† , African traditional religion, Islam.

‡ , Senior, principal, chief registered nurses, and nursing officers.

§ , Community healthcare nurse, family healthcare practitioner.

### Clinical characteristics and prevalence of attitudes

Only 10.9%^22^ of respondents have a personal history of mental illness. The majority, 59.9% (124), do not have any experience of working in a psychiatric unit. Over half of them, 51.5% (104), had a negative attitude towards people with mental illness, using the mean MICA attitude score ≥ 41.79 as the cut-off point (Table 2).

**TABLE 2:** Clinical characteristics and attitude of the 202 primary healthcare nurses.

Characteristic	Frequency	%
**Personal history of mental illness**	**201**	**100.0**
Yes	22	10.9
No	179	89.1
**Know someone with mental illness**	**200**	**100.0**
Yes	187	93.5
No	13	6.5
**Family history of mental illness**	**201**	**100.0**
Yes	45	22.4
No	156	77.6
**Experience of working in a psychiatric unit**	**201**	**100.0**
Yes	77	40.1
No	124	59.9
**Attitude**	**202**	**100.0**
Negative†	104	51.5
Positive‡	98	48.5

Note: Bold values indicate the total of participants who responded and frequency.

† , Total attitude score of ≥ mean (41.79).

‡ , Total attitude score of < mean score (41.79).

### Relationship between the outcome and categorical variables

Nurses who were not married were likely to hold negative attitudes towards people with mental illness (*t* = 2.491; *p* = 0.014). General nurses (who were not specialised) were also likely to have negative attitudes (*t* = 3.220; *p* = 0.002). Being a non-psychiatric nurse was associated with negative attitudes (*t* = 2.215; *p* = 0.028). Those with a personal history of mental illness were more likely to have negative attitudes (*t* = 3.745; *p* < 0.01). Nurses who have experience working in a psychiatric unit were more likely to have positives attitudes compared to those without that experience (*t* = 2.706; *p* = 0.007) (Table 3). On Table 4, knowledge was found to be negatively correlated with attitudes.

**TABLE 3:** Relationship between the outcome and categorical variables.

Variable	*N*	Mean	s.d.	*t*-test	*p*
**Sex**					
Male	76	42.8	9.4	1.208	0.229
Female	126	41.2	9.8	-	-
**Health Facility**					
Hospital	113	42.6	9.6	1.399	0.163
Clinic or health post	89	40.7	9.7	-	-
**Marital status**					
Not married	146	42.8	9.6	2.491	**0.014**
Married	56	39.1	9.4	-	-
**Educational qualification**					
Diploma	159	42.4	9.8	1.516	0.131
Degree	41	39.8	10.1	-	-
**Designation**					
Registered nurse	82	42.3	8.7	0.757	0.450
Above registered†	119	41.2	10.0	-	-
**Speciality**					
General nurse	164	42.9	9.4	3.220	**0.002**
Specialised nurses‡	35	37.2	9.8	-	-
**Psychiatric and non-psychiatric nurses**					
Non psychiatric	193	42.2	9.7	2.215	**0.028**
Psychiatric	6	33.3	4.7	-	-
**Personal history of mental illness**					
Yes	22	48.6	7.2	3.745	**0.000**
No	179	40.8	9.4	-	-
**Know someone with mental illness**					
Yes	187	41.4	9.4	1.495	0.136
No	13	45.5	11.3	-	-
**Family history of mental illness**					
Yes	45	43.8	7.7	1.690	0.93
No	156	41.1	9.9	-	-
**Experience of working in a psychiatric unit**					
Yes	77	43.9	8.7	2.706	**0.007**
No	124	40.2	9.7	-	-

Note: Bold values indicate the total of participants who responded and frequency.

s.d., standard deviation.

† , Senior, principal, chief registered nurses, and nursing officers.

‡ , Midwives, psychiatric, community health, family nurse practitioner.

Significant *p*-values are in bold.

**TABLE 4:** Correlation between the continuous variable and the outcome.

Variables		1	2	3	4	5
1	Attitudes	1.000	−0.074	−0.133	−0.059	−0.186**
2	Age	-	1.000	0.263**	0.956**	0.133
3	Income	-	-	1.000	0.255**	−0.041
4	Years of experience	-	-	-	1.000	0.072
5	Knowledge score	-	-	-	-	1.000

** , Correlation is significant at the 0.01 level (2-tailed).

### Factors associated with attitudes

Factors that were significantly associated with attitudes on both bivariate analyses were further included in the multiple regression model to test for their associations with the outcome. Only three factors (speciality, personal history of mental illness, and knowledge score) remained significantly associated with attitudes. General nurses were more likely to have negative attitudes towards people with mental illness (*B* = –0.184; *p* = 0.014). Those with a personal history of mental illness were more likely to develop negative attitudes (*B* = –0.215; *p* = 0.002). Nurses with less knowledge about mental illness were also more likely to have negative attitudes (*B* = –0.149; *p* = 0.032) (Table 5).

**TABLE 5:** Multiple regression analyses of variables associated with attitudes (outcome).

Variables	*B*	*t*	*p*
Marital status	−0.106	−1.546	0.124
Speciality	−0.184	−2.483	**0.014**
Personal history of mental illness	−0.215	−3.174	**0.002**
Psychiatric nurses	−0.051	−0.708	0.480
Knowledge score	−0.149	−2.163	**0.032**
Experience of working in a psychiatric unit	0.093	1.318	0.189

Note: Significant *p*-values are in bold.

## Discussion

We set out to assess the attitudes of nurses working in a primary healthcare setting (Greater Lobatse health district) towards people with mental illness. Our findings revealed that 51.5% of primary healthcare nurses have negative attitudes. Also, the nurses’ speciality, personal history of mental illness, and knowledge score remained significantly associated with attitudes.

The 51% prevalence of negative attitudes in this study is consistent with results from a similar survey conducted in Ethiopia, which reported that nearly 50% of the primary healthcare nurses had negative attitudes towards people with mental illness.^14^ Nonetheless, our study stands in contrast to other previous investigations in Africa, as they report variances in the prevalence rates of predominantly negative attitudes^8,18,39,40^ and positive attitudes towards the mentally ill.^17,41^ The prevalence of negative attitudes towards mental illness among nurses in African countries may, to some extent, be attributed to prevailing societal attitudes towards people with mental disorders.^15^ In many African societies, mental illness is viewed as a result of evil machinations, which often leads to societal stigma and, in turn, affects nurses’ attitudes towards mental illness. This finding highlights the need for further research to explore the underlying factors contributing to the disparate rates of negative attitudes across the region. Such research could provide valuable insights for policymakers and healthcare practitioners seeking to improve mental health outcomes in African countries.

Also, the findings of this study reveal negative attitudes among nurses towards people with mental illness, which are in contrast to the mixed prevalence rates and differing attitudes observed globally.^10^ Worldwide, the attitudes of nurses towards individuals with mental illness tend to be varied. This variation may be attributed, in part, to the views held by different societies. For example, while most European nurses held positive attitudes, most African and Asian nurses held negative attitudes. Furthermore, the mixed attitudes of nurses across the globe could be because of the use of different measures and tools with varying psychometric properties, yielding differing results.^10^

The prevalence of negative attitudes, as demonstrated by this study, among primary healthcare nurses is a matter of great concern. This is particularly worrisome as it can have an impact on the delivery of mental healthcare services. Negative attitudes among healthcare professionals have been linked to poor management outcomes for individuals with mental illness, resulting in decreased willingness to access healthcare services, lower rates of treatment adherence, and reduced chances of recovery.^8^ In the light of these findings, there is a pressing need for anti-stigma interventions aimed at addressing and changing the negative attitudes of nurses towards mental illness. Such interventions can have a significant impact on the delivery of mental healthcare services and improve patient outcomes.

Our study revealed that negative attitudes were associated with being a non-specialised nurse, having less knowledge about mental illness, and having a personal history of mental illness. Those who were general nurses were more inclined to have negative attitudes compared to those who were specialised. This finding is supported by the results of studies conducted in Ethiopia and the Western Cape in South Africa.^14,39^ In line with this study, the above-mentioned studies reveal that nurses with higher educational qualifications tend to have more positive attitudes compared to those with lower educational status. This is most probable because professionals with post-basic training have opportunities to have more theoretical and practical knowledge about mental illness; these factors could contribute to their positive view of people with mental illness.^14^

The finding that less knowledge about mental health is associated with negative attitudes is consistent with the findings of studies conducted in Jamaica and Ethiopia.^14,42^ These similar studies found that nurses with no mental health training endorsed stigmatising attitudes towards people with mental illness. Sahile and colleagues found that the odds of having negative attitudes were 2.83 times higher in those with basic medical knowledge compared to those nurses with good or advanced knowledge of mental health.^14^

Furthermore, it has been established that poor knowledge is one of the contributory factors to stigma^43^; hence, it can be argued that more programmes aimed at improving mental health knowledge are much-needed interventions in order to improve nurses’ attitudes and, ultimately, enhance mental healthcare delivery. One method of empowering healthcare professionals with the necessary knowledge and skills to provide effective mental healthcare is through periodic continuing medical education for those who are already practising. In addition, enhancing the nursing training curriculum to include a greater focus on mental healthcare could provide nurses with more comprehensive knowledge and practical experience in this field. Other settings, such as South Africa and Ethiopia, have found these effective in addressing poor mental health knowledge and negative attitudes.^14,44^

Personal history of mental illness refers to individuals who have or have had a mental illness themselves. Personal history of mental illness predicted negative attitudes among respondents of this study. This finding is in contrast with a survey carried out in Germany, which found that people with a personal history of mental illness tend to react more prosocially, develop feelings of anxiety less frequently, and express less desire for social distance.^45^ Corrigan and colleagues also found that familiarity with mental illness diminishes prejudice towards people with mental illness; this is against our findings.^46^ It is generally expected that personal experience or familiarity improves knowledge of mental health and, hence, attitudes.^45^

One possible explanation for our above-mentioned finding could be projective identification as a defence mechanism. Melanie Klein defined projective identification as the transference of psychological content from one person to another by externalising part of the self onto an external object.^47^ This allows a person to experience feelings as more bearable and reduces anxiety.^47^ In consideration of this theory, the nurses in our study could have had feelings of shame about their mental illness because of internalised stigma, and they used the defence mechanism of projective identification to protect themselves against this feeling of shame. This could have translated into measured negative attitude.

Notwithstanding this finding, one study conducted among Indonesian nursing students found that personal history of mental illness did not correlate with negative attitudes towards those with mental illness.^48^ The finding of this study with regard to personal history and negative attitudes further emphasises the fact that education about mental illness promotes positive attitudes.

### Strengths and limitations

When interpreting the results of this study, it is important to note certain limitations. The cross sectional nature of the study and convenience sampling limits the generalisability of the findings. Nonetheless, to the best of our knowledge, this is the first study to assess nurses’ attitudes towards people with mental illness in Botswana, hence it provides a starting point for research on this topic in this setting. It is suggested that future studies could address the methodological limitations of this study. Another limitation, which is also one of the main debates in literature, is that the measured attitudes do not necessarily translate to behaviour. Nevertheless, results from one study suggest that prejudicial attitudes have a direct influence on behaviour.^49^

The tool used to assess knowledge, MAKS, does not assess general mental health knowledge per se, but rather stigma-related mental health knowledge. It should, therefore, be interpreted with caution and in conjunction with other stigma measures as advised by the authors of the tool. In this study, it was used in conjunction with the MICA-4 tool for attitudes. This gives an idea of the effect of the lack of mental health knowledge on attitudes.

### Implications on practice and future research

There should be in-service training in basic mental health knowledge, targeting nurses at primary healthcare facilities as they play a key role in managing patients with mental health problems. In addition, there should be an initiation of special anti-stigma campaigns aimed at raising awareness about mental health issues and decreasing stigmatising attitudes among the general public and important groups, including primary healthcare professionals.

Future studies with improved methodological approach are recommended to add more insights about the attitudes of nurses towards people with mental illness in Botswana.

## Conclusion

The aim of the study was to determine the attitudes of nurses towards the mentally ill and associated factors in a primary healthcare setting. More than half of the respondents, 51.5%, have negative attitudes towards people with mental illness. The factors associated with these negative attitudes were being a non-specialised nurse, having less mental health knowledge, and having a personal history of mental illness. The findings emphasise the need to initiate training and educational programmes that aim to reduce stigma and increase mental health knowledge among primary healthcare nurses. This will be important in improving mental healthcare delivery in Botswana and in line with the WHO recommendation of integrating mental health services into primary healthcare systems.
